# Atrial Bigeminy, a Potential Diagnostic Clue for Glioblastoma

**DOI:** 10.15190/d.2024.4

**Published:** 2024-03-31

**Authors:** Cristina Mihaela Stirbu, Daniel Teleanu, Mircea Furtos, Teodora Ghica, Ruxandra Dragoi Galrinho

**Affiliations:** ^1^Military Medicine Institute, 3-5 Institutul Medico-Militar Street, 010919, Bucharest, Romania; ^2^Carol Davila University of Medicine and Pharmacy, 8 Eroii Sanitari Boulevard, 050474, Bucharest, Romania; ^3^Neurosurgery Department, Emergency and University Hospital of Bucharest, 169 Splaiul Independentei, 050098, Bucharest, Romania; ^4^Cardiology and Cardiovascular Surgery Department, Emergency and University Hospital of Bucharest, 169 Splaiul Independentei, 050098, Bucharest, Romania

**Keywords:** Glioblastoma, Atrial Bigeminy, Syncope; Bradycardia, Cerebral Tumor.

## Abstract

Glioblastoma represents the most common and aggressive primary malignant central nervous system tumor, often manifesting with unusual signs. This case report highlights a patient diagnosed with glioblastoma following an unusual cardiac presentation, with syncopes, sinus bradycardia, and atrial bigeminy. A 51-year-old female, brought to the emergency room after experiencing repeated syncope episodes, displayed neurological deficits upon examination. Noteworthy, she presented abnormal ECG showing sinus bradycardia and atrial bigeminy. Following the diagnostic procedure, a tumor was identified with indication to surgical removal. A subtotal tumor resection was obtained and the morphopathology examination led to a glioblastoma diagnosis. Interestingly, post-operatively, the ECG was completely normalized. However, the patient experienced complications, consisting of a massive thromboembolism. While sporadic cases describe unusual glioblastoma manifestations, this report is unique in showcasing atrial bigeminy, among other ECG manifestation. The remission of atrial bigeminy post-operatively suggests its association with the glioblastoma. Tumor localization in the basal ganglia is crucial in understanding such manifestations. Idiopathic cardiac manifestations should not be disregarded, holding potential relevance in central nervous system etiology considerations.

## SUMMARY

1. Introduction

2*. *Case Report

3*. *Important findings of this clinical case for the clinical/scientific community

4. Discussion

5. Conclusion

## 1. Introduction

Glioblastoma^1^ is the most common and aggressive type of primary malignant central nervous system tumor^2^. Besides neurological symptoms, patients may exhibit atypical manifestations associated with various visceral sites^3^. We present the case of a patient who was diagnosed with glioblastoma subsequently suffering repeated syncopes, sinus bradycardia and atrial bigeminy. Glioblastoma rapidly occupies space within the brain causing a mass effect, thus increasing intracranial pressure and further leading to certain signs, such as bradycardia and aphasia like in this patient’s case.

## 2. Case report

A 51-year-old female patient was brought by ambulance to the emergency room with an altered state, after experiencing repeated syncope episodes. Her state was assayed through Glasgow Coma Scale, which was of 14 points. She was still confused, and the neurological examination revealed left hemiparesis, hypotonia and mixed aphasia. She was admitted with a Karnofsky Performance Scale of 70 points. Notably, six months prior to this event the patient has done a Magnetic Resonance Imaging (MRI) in another medical service, which did not highlight any anatomical alterations.

**Figure 1:**
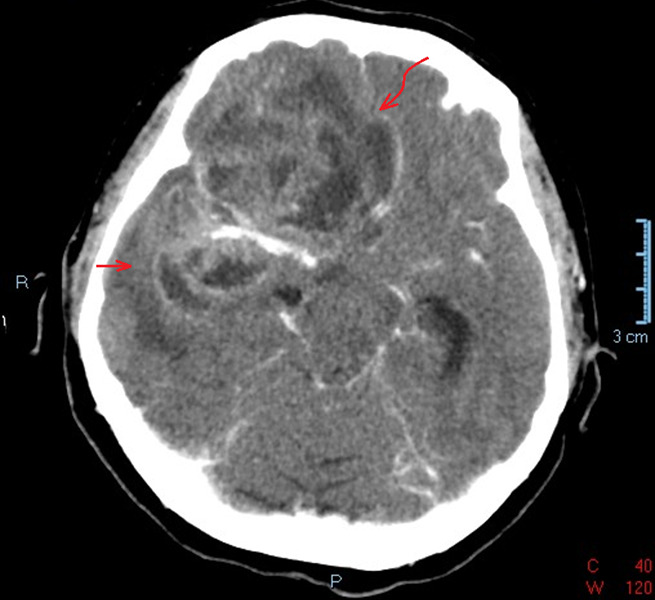
Preoperative CT The tumor is highlighted invading the frontal and temporal lobes as well as the head of the caudate nucleus, the internal capsule and the lenticular nucleus, surrounded by edema (straight arrow) and shifting the midline (undulated arrow).

At that time, she complained of severe cephalalgia, lethargy and vertigo. A computed tomography (CT) done in the treating facility, revealed an expansive mass in the right frontal and temporal lobes, invading the head of the caudate nucleus, the internal capsule and the lenticular nucleus (Figure 1) and edema, shifting the midline with 10 mm to the left hemisphere.

**Figure 2:**
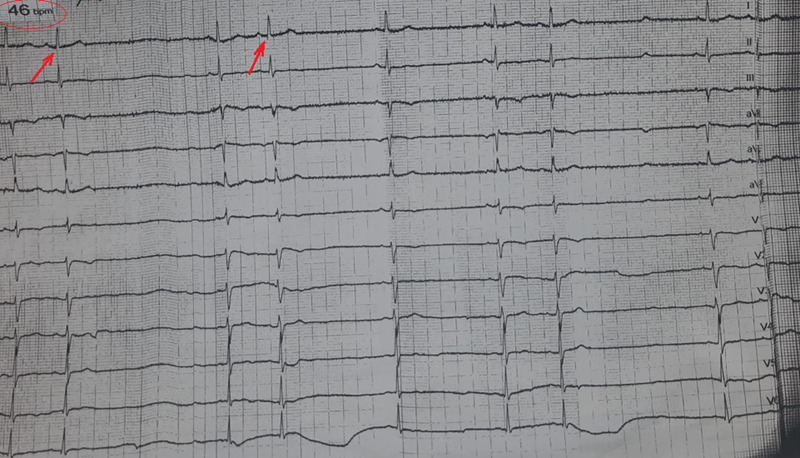
Patient’s ECG preoperatively The ECG done before surgery displays sinus bradycardia of 46 bpm (circled in red) and atrial bigeminy (arrows).

Additionally, the routine electrocardiogram (ECG) was abnormal, showing sinus bradycardia, alternating with atrial bigeminy (Figure 2). Moreover, the cardiac clinical exam and echocardiogram were normal. Considering the entirety of the clinical and paraclinical data, this patient’s pathology presented indications for neurosurgical intervention. The tumor was subtotally resected, using a large right pterional craniectomy. It was decided upon removal of the bone flap due to persistent intraoperative brain swelling and high chances of tumor recurrence even more aggressively.

**Figure 3:**
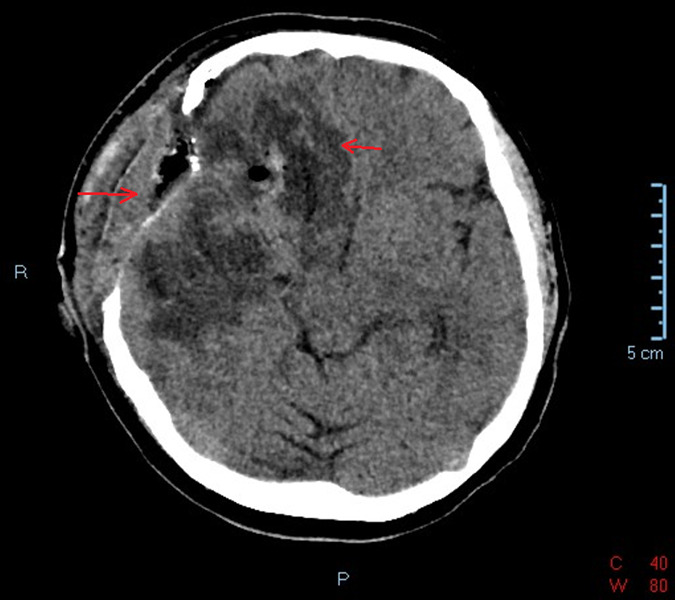
Postoperative CT This CT illustrates the subtotal resection of the tumor along with intracranial aerocele, edema and inflammation.

The morphopathology examination led to the final diagnosis of glioblastoma or grade IV astrocytoma, both on the frozen section and formalin-fixed, paraffin-embedded section. Post-operatively, the CT scan unveils the space once occupied by the tumor, intracranial aerocele, edema and inflammation. All these aspects were considered normal in these circumstances (Figure 3). Noteworthy, the cardiac rhythm was normalized, so a cardiology exam was not considered necessary. Epilepsy episodes were taken into consideration, but the patient’s family denied seeing any abnormal events, nor were any observed during admission. Nevertheless, taking into consideration the localization and the extent of the mass, prophylaxis with Phenytoin anticonvulsants was started. Despite the challenges, she regained most of her motor functions, remained alert and spatially and temporally oriented. The patient was then discharged and referred to an oncology department, where she would undergo appropriate treatment for her diagnosis.

**Figure 4:**
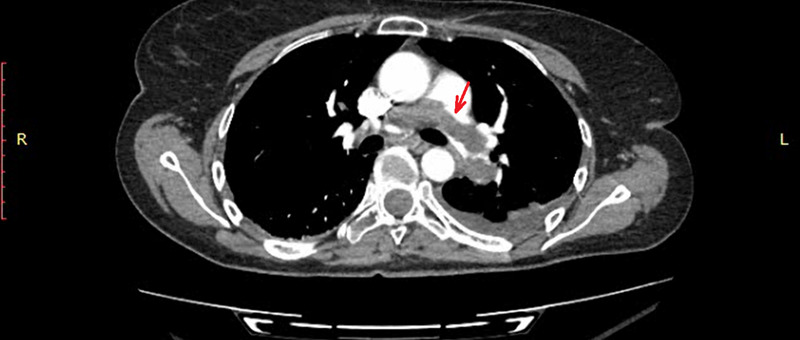
Thoracic CT scan with contrast It showed a massive pulmonary thromboembolism in both pulmonary arteries, having a saddle-like appearance (arrows).

Unfortunately, three weeks later, the patient encountered complications. She suffered deep vein thrombosis and intermediate-high risk pulmonary embolism, with a Pulmonary Embolism Severity Index (PESI) score of 121 points. Putatively these complications arose from the high thrombogenic potential^4,5^ of this type of cancer and her being immobilized in a dorsal decubitus position postoperatively, in respect to the medical recommendations given. Her thoracic CT scan showed a massive pulmonary thromboembolism in both pulmonary arteries, having a saddle-like appearance (Figure 4).

**Figure 5:**
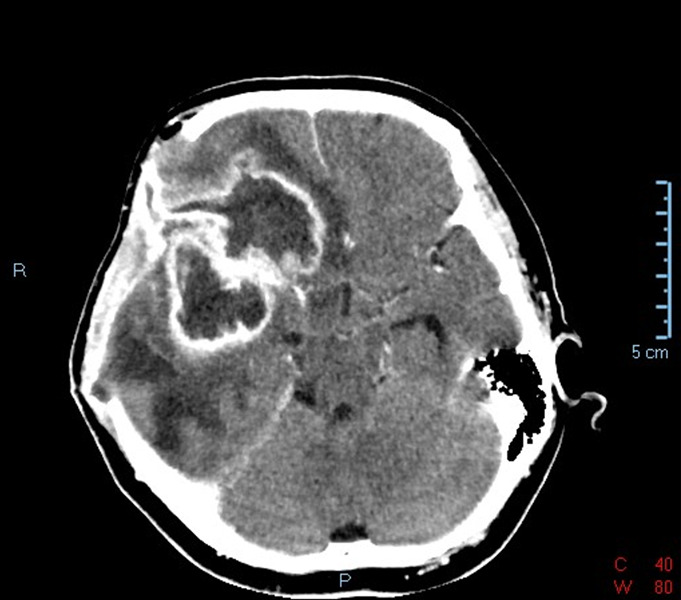
The cerebral CT done during the second admission It shows enlargement of the remaining tumor postoperatively and reduced inflammation.

**Figure 6:**
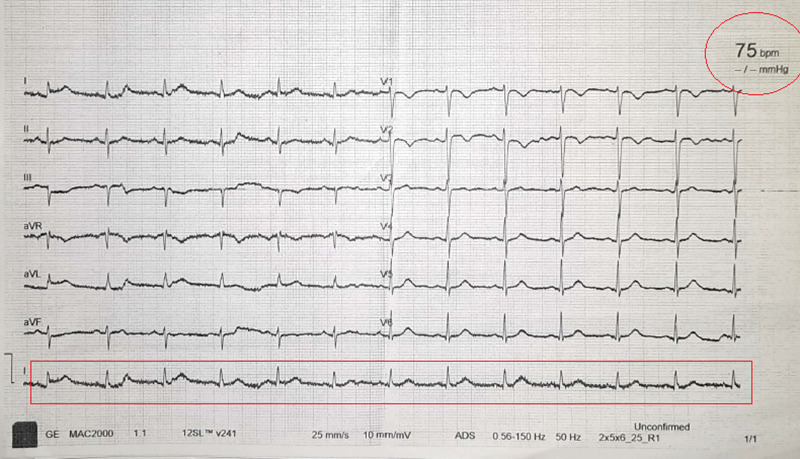
Patient’s ECG on second admission This ECG displays normal sinus rhythm of 75 bpm (circled).

Thrombolysis was contraindicated due to her recent surgery and persistent central nervous system neoplasm, therefore she was prescribed low molecular weight heparin (LMWH) in therapeutic dose during hospitalization, switched to Apixaban at discharge. In addition, the remaining tumor had enlarged drastically as shown in the check up cranial CT (Figure 5). As shown in this ECG, the patient no longer displayed sinus bradycardia, nor atrial bigeminy (Figure 6). She was discharged after 12 days inwards.

## 3. Important findings of this clinical case for the clinical/scientific community

- Idiopathic abnormal cardiovascular manifestations should be explored further, with consideration given to a central nervous system etiology;

- The high thrombogenic potential of glioblastoma should not be overlooked when determining the appropriate treatment for thrombosis.

## 4. Discussion

Unusual signs of glioblastoma have been sporadically described in literature. Nonetheless, no other case reports were found in literature showcasing atrial bigeminy as an important manifestation of glioblastoma.

Considering the remission of the bradycardia, syncopes and atrial bigeminy postoperatively, it is suggested that the glioblastoma may have contributed to this phenomena. The presumed cause of the syncope episodes was bradyarrhythmia induced by the glioblastoma’s space-occupying feature, thus increasing intracranial pressure. This was not electrically emphasized, as the patient did not experience any other syncope episodes preoperatively, and no cardiac conduction disturbances were observed. Albeit bradycardia is an important element of the Cushing’s triad used for assessing increased intracranial pressure of different etiologies^^6^^, it is believed that its association with atrial bigeminy and syncope episodes in this patient was not inadvertent. Nonetheless, the lack of consensus in literature regarding the time framing of the cardiovascular and respiratory adverse effects correlated with intracranial masses was observed, whether they are early signs^^7^^ or appear after neurological or psychiatric impairments^^8^^. It is not incorrect to assume that surrounding areas were affected by this tumor and thus leading to these manifestations. For instance, Deep Brain Stimulation (DBS) targeting the claustrum in animal models has revealed an increased vagal activity, which could ultimately lead to parasympathetic expression^^9^^. The claustrum is an anatomical structure in close proximity to the basal ganglia^^10^^ and it was most definitely affected in this patient’s case.

Similarly, a patient with a history of repeated syncope episodes and bradycardia was diagnosed with an anaplastic astrocytoma localized in the left basal ganglia^^11^^. Interestingly, two cases of glioblastoma manifesting with epileptic seizures^^12^^ were reported in association with syncope episodes and bradycardia, which played leading roles towards diagnosis^^3,13^^. Furthermore, a few cases documented syncope as the primary manifestation, often associated with headaches, hemiparesis, hemihypoesthesia, nausea, or vomiting^^14,15^^.

The localization within the basal ganglia is imperative for this article, given that the focal point of the discussed tumor was identified in the right dorsal striatum as well as the frontal and temporal lobes. Moreover, it was noted that the neoplasm's specific location constituted a shared characteristic between this case report and the referenced cases.

## 5. Conclusion

Further investigation is warranted to explore the relationship between syncopes, sinus bradycardia and atrial bigeminy with intracranial space-occupying expansive processes, elucidating whether it serves as a direct cause of the malignant mass or if it is secondary, potentially arising from increased intracranial pressure and subsequent systemic distress. Additionally, it is crucial not to overlook idiopathic cardiac manifestations, as they may hold relevance in considering a central nervous system etiology.

## Bullet Points

*- The high thrombogenic potential of glioblastoma should not be overlooked when determining the appropriate treatment for thrombosis.*

*- Idiopathic abnormal cardiovascular manifestations should be explored further, with consideration given to a central nervous system etiology.*
